# Performance monitoring and accountability: The Agile Project’s protocol, record and experience

**DOI:** 10.12688/gatesopenres.13119.2

**Published:** 2020-08-20

**Authors:** Amy Tsui, Philip Anglewicz, Titilope Akinlose, Varsha Srivatsan, Pierre Akilimali, Souleymane Alzouma, Fiacre Bazie, Peter Gichangi, Georges Guiella, Patrick Kayembe, Anupam Mehrotra, Funmilola OlaOlorun, Elizabeth Omoluabi, Sani Oumarou, P. R. Sodani, Mary Thiongo, Meagan Byrne, Kurt Dreger, Michele Decker, Carolina Cardona, Pierre Muhoza, Carolyn Combs, Alain K. Koffi, Scott Radloff

**Affiliations:** 1 Department of Population, Family and Reproductive Health, Johns Hopkins Bloomberg School of Public Health, Baltimore, MD, 21205, USA; 2 University of Kinshasa, Kinshasa, Democratic Republic of the Congo; 3 Institut National de la Statistique, Niamey, Niger; 4 Higher Institute of Population Sciences, Joseph Ki-Zerbo University, Ouagadougou, Burkina Faso; 5 International Centre for Reproductive Health Kenya, Nairobi, Kenya; 6 IIHMR University, Jaipur, India; 7 University of Ibadan, Ibadan, Nigeria; 8 Centre for Research, Evaluation Resources and Development, Ife, Nigeria; 9 Department of International Health, Johns Hopkins Bloomberg School of Public Health, Baltimore, USA

**Keywords:** Family planning, service delivery, clients, supply, quality, consumption

## Abstract

The Performance Monitoring and Accountability 2020 (PMA2020) project implemented a multi-country sub-project called PMA Agile, a system of continuous data collection for a probability sample of urban public and private health facilities and their clients that began November 2017 and concluded December 2019.  The objective was to monitor the supply, quality and consumption of family planning services.  In total, across 14 urban settings, nearly 2300 health facilities were surveyed three to six times in two years and a total sample of 48,610 female and male clients of childbearing age were interviewed in Burkina Faso, Democratic Republic of Congo, India, Kenya, Niger and Nigeria.  Consenting female clients with access to a cellphone were re-interviewed by telephone after four months; two rounds of the client exit, and follow-up interviews were conducted in nearly all settings.  This paper reports on the PMA Agile data system protocols, coverage and early experiences.  An online dashboard is publicly accessible, analyses of measured trends are underway, and the data are publicly available.

## Introduction

When monitoring and evaluating (M&E) the performance, progress and impact of large-scale population-level interventions, the standard practice in developing country settings has been to rely on cross sectional surveys conducted by third parties usually at the beginning and the end of the project to provide information on changes in outcomes of interest. These specialized surveys are usually extensive in scope and rich in detail and instrumental for global monitoring ( Boerma & Sommerfelt, 1993), but their deployment is also resource- and time-intensive. When findings from these surveys become available, their dissemination is often much later than needed to modify the design or continued implementation of the project. Moreover, it is often necessary to relate the findings with contextual information from other data sources in order to gain insights on why and how the project succeeded or not. In short, the traditional M&E approach is not designed for tracking and acting on performance results on an ongoing basis ( Nordberg, 1988; Rowe, 2009).

At the same time, health information systems, while increasingly digitized, are constrained in the types of measures available, their selective coverage of facilities and clients, accuracy of gathered data, and timeliness of reporting. Efforts to eliminate these deficiencies are growing, especially to address prevention and control of large-scale epidemics of infectious diseases ( Braa *et al.*, 2007; Walsham & Sahay, 2006). The lack of systematic and programmatically relevant, continuous and timely information available at subnational levels, however, has posed a formidable challenge to nimble and effective project decision-making and response (see Guenther *et al.*, 2014; Maina *et al.*, 2017). PMA Agile was designed to move away from the traditional M&E approach by establishing a near-continuous monitoring system that collects, links and aggregates data at different levels on a focused set of indicators in a cost-effective manner. The Agile system was developed to reduce the lag time between steps in a project learning process: recognizing a project’s information needs, identifying sensitive performance indicators, collecting relevant primary and secondary data, analyzing the collected data, producing actionable insights, and enabling the use of the insights to adjust and fine-tune programs.

PMA Agile evolved out of a combined interest of the research and evaluation staff at the Bill and Melinda Gates Foundation and the PMA ^1^ project at the Johns Hopkins Bloomberg School of Public Health to develop an innovative data system that could track performance of two large projects ^2^ at the subnational level, in this case urban areas and their poor. In Agile’s first year, the selection of urban geographies for monitoring was dependent on these two projects’ own plans for locating their program resources. It later became clear that Agile would need to proceed more independently with geographic selection, than originally planned in order to realize its objectives in the awarded project period. The eventual selection of Agile geographies did not affect its design or four objectives which are to:

1. Provide a flexible, continuous and cost-effective data collection system that can triangulate with routine and other survey information;
2. Serve as an adaptable, replicable M&E platform for program implementation addressing needs of the urban poor;
3. Measure core indicators that reflect program performance at the health facility and client levels;
4. Promote actionable findings to enable evidence-based decisions by government officials, non-government stakeholders and researchers.

This paper describes the protocols, record and experiences, to date, of PMA Agile to accompany the findings that are already available on public dashboards. Because the data will become publicly available, this description provides important background to inform current and future users.

## Protocol

### Urban settings for PMA Agile

Cities have become home to growing, underserved poor communities. More than half of the world’s population currently lives in cities and this urbanization is accelerating to 70 percent by 2050, especially in Africa and Asia ( United Nations, 2019). Cities benefit from economic growth but their governments struggle to accommodate rising demands for services. Reaching urban women and girls with reproductive health services has become a social welfare imperative.

Cities also offer the advantages of spatial proximity to health services with transportation systems, population density, and structural networks among individuals and institutions to support a private health sector that ranges in type from retail kiosks and pharmacies dispensing over the counter medications to specialist doctors and hospitals that cater to more elite client needs. To monitor the implementation of any large-scale health initiative aimed at reducing urban disparities and improving health equity requires gathering information on the private, as well as public, health sectors. Social marketing projects largely target urban populations to reach a market that enables subsidizing commodities for the poor. They function by providing a range of high-quality, affordable, and novel brands of contraceptive and sexual health products to the market through well established distribution and supply chain mechanisms.

### Selection of PMA Agile sites

PMA Agile activities officially began November 2016 with the first set of five country and 12 city geographies decided in July 2017: Burkina Faso (Ouagadougou and Koudougou); Democratic Republic of Congo (DRC) (Kinshasa); India (Ferozabad, Indore, Puri and Shikohadbad/Tundla cities); Kenya (Kericho, Migori and Uasin Gishu counties); and Nigeria (Lagos, Ogun and Kano). PMA Agile site location was based on collaborating with existing PMA implementing partners and considerations of local intervention activities, government health administration interest and willingness to act on results and input from Gates Foundation staff. Capitals of two Francophonic countries (Abidjan in Cote d’Ivoire and Niamey in Niger) were held for future consideration, with Niamey subsequently added in early 2019 as the 14 ^th^ PMA Agile site. 

### Implementing partners (IPs)

IPs’ capacity and connections with local stakeholders were key for the successful dissemination and actionability of PMA Agile results. The implementing partners were the Center for Research, Evaluation Resources and Development and the University of Ibadan for Nigeria, the Indian Institute for Health Management Research in India, the Institut National de Statistiques of the Government of Niger, the Institut Superieur de Sciences de la Population in Burkina Faso, the International Center for Reproductive Health in Kenya, and the University of Kinshasa School of Public Health for DRC.

### Data elements

PMA Agile’s data system has four main elements: a baseline and quarterly follow-up health facility survey, a semi-annual client exit interview survey of male and female clients, a follow-up phone interview of consenting female clients, and a youth survey based on respondent-driven sampling. Table 1 provides an overview of each element’s purpose, mode of administration, sample, eligibility criteria, target sample size and periodicity ^3^.

**Table 1.** Features of elements for PMA Agile data system.

Element	Purpose	Mode of administration	Sample	Eligibility criteria	Target sample size	Periodicity
Health facility survey	Measure availability and status of key indicators of contraceptive service delivery	Face to face interview with facility manager or knowledgeable informant	Probability sample of urban public and private health facilities	Registered health facilities	220 public and private per Agile site	Quarterly
Client exit survey	Service experience and satisfaction	Face to face interview	Systematic sample of clients using facility services	Female clients age 18 to 49 and male clients age 18 to 59 upon completion of visit	10 clients per selected facility	Semi-annual
Client follow-up survey	Measure any change in contraceptive status and satisfaction with services	Telephone follow-up	Female clients	Baseline clients who consent and provide phone number(s)	All eligible clients	Semi-annual (in following quarter)
Youth respondent driven sample (YRDS)	Measure contraceptive procurement among youth	Computer-assisted self-administered interview	Respondent driven sample in 3 selected urban cities	Unmarried females and males age 15 to 24 recruited by seeds	Abidjan - 2000 Nairobi - 1300 Lagos - 1300	One time

## Sample selection and size

### Health facility or Service Delivery Point (SDP) ^4^

Respondents for the SDP questionnaire were primarily the in-charge/manager of the health facility; however, once the respondent has given consent for the SDP to participate, other personnel at the facility occasionally contributed answers based on expertise/knowledge of the subject matter. These other respondents may include medical staff, pharmacists or accountants. All SDPs that participate in the baseline SDP survey become eligible for subsequent quarterly follow-up surveys.

The size of the SDP sample was determined using the proportion that provides three or more contraceptive methods. In Kenya, the first PMA Agile site, this proportion was 77% of SDPs based on data from five earlier rounds of PMA surveys. With 80% power, alpha of 0.05, and allowing for a 5.5% margin of error, the required simple random sample size was 204 health facilities. After allowing for 15% non-response, the sample size for SDPs was fixed at 220 across all Agile sites and evenly divided into 110 public and 110 private facilities. Lists of registered public and private health providers were obtained from relevant official authorities. The lists included facility names, type of facility and addresses. The facilities were stratified by public and private and then the proportionate distribution of facility type was calculated. The 110 facilities in each sector were then randomly selected. If a site had fewer than 110 facilities, all facilities in that sector were selected to be surveyed ^5^. This panel of SDPs was then visited quarterly for follow-up surveys. Preliminary field checks were made to assess the accuracy of the lists but more often, if a sampled facility at baseline was found to be non-existent, closed or transformed into another type of facility, it was replaced with another facility of the original type drawn from the list. 

Mobile-phone based survey forms, akin to those used by PMA (see Zimmerman *et al.*, 2017), were developed to consent and interview the in-charge or owner of the health facility on a quarterly basis. The baseline questionnaire or form is about 30 minutes in duration with the quarterly follow up about 15–20 minutes. Consent rates for baseline and retention rates for continued quarterly survey participation have been relatively high across sites, as will be seen below. Incentives were not given to SDP survey participants. However, in Nigeria, retention of the participation of private health facilities over time required providing an additional incentive (a PMA Agile-branded wall clock).

### Client exit interviews

The CEI survey was aimed at capturing the service experiences of adults seeking ambulatory health care. It targeted interviews with 10 clients per sampled facility. This number is based on a sample power calculation using a modern contraceptive prevalence of 50%, assumed to be fixed across all Agile sites, a margin of error of 3% and design effect of 2. This resulted in a sample size of 2106 clients which divided by 220 health facilities resulted in 10 clients per facility.

Eligibility criteria for the CEIs were: female clients 18 to 49 years old or male clients age 18 to 59 years. Clients were recruited systematically or sequentially by the field interviewer (known as the resident enumerator or RE) as they exit the sampled SDPs over the course of one or two interview days. The RE was provided the average daily client volume for the SDP, obtained during the baseline survey and a sampling interval. For example, if the SDP saw an average 150 clients per day, the RE was given a sampling interval of 15 to select 10 clients. The RE used a random start number between 1 and 15 and began recruitment with the Nth client who exited. REs worked in pairs at large health facilities, such as hospitals, and also position themselves at the outpatient and primary care clinics for survey recruitment. At small facilities, such as pharmacies, the same systematic selection procedures were followed, and REs could work in pairs depending on client volume. CEIs were generally completed outside the pharmacies. 

Clients were approached to participate after they received or attempted to receive care from the SDP. Trained team enumerators introduced themselves, explained the Agile survey to clients and consented the client to participate. Clients consenting and completing the survey were provided with $1 equivalent in cell phone airtime or offered a material good of equivalent value as compensation for their time.

The CEI was approximately 20 minutes in length and collected information on client experience and satisfaction with the health site’s services, with family planning content prioritized. The CEI was fielded in the second and fourth quarterly surveys (Q2 and Q4) each year, or two times over a 12-month period. Participation rates (the percentage of clients consenting to be interviewed) were above 50% in all settings and ranged from 4% non-consent in Kenya to 35% non-consent in Nigeria among female clients (data not shown).

### CEI Follow-Up

To assess contraceptive practice, only female clients were recruited for the CEI phone follow-up survey. Upon completion of the CEI, the female client was asked if she was willing to participate in a follow-up interview to occur in approximately 4 months. If she consented, she was asked for a primary and secondary phone number (cell or landline) at which she could be reached. Often female clients provided their male partners’ phone numbers and the script used at the beginning of the call was general to avoid disclosing any confidential health behavior.

The phone follow-up interview asked about the female’s adoption (among those who were not using a method at the time of the CEI) and continued use or switching of contraceptive methods and continued satisfaction with the health facility visited. The four-month interval was selected to enable re-supply of short-term methods such as the three-month injectable and to optimize on retention of the client sample. In the absence of much published literature on participation rates for follow-up surveys administered by telephone in developing country settings, it was expected that approximately half of the client sample would be female and that half would consent to the phone follow-up, leading to approximately 500 clients re-interviewed. In actuality, the average proportion of CEIs with females across the 14 sites and all rounds was 64.7% and was highest in Niamey, Niger at 92.5% and lowest at 32.1% in Puri, India. Follow-up participation rates ranged from 37.3% in Shikohadbad-Tundla, India to 96.6% in Migori, Kenya, with an average of 70.2%. **

The RE team set aside specific days to conduct the CEI follow-up in a project office. They were provided their individual list of consenting females, typically ones they had interviewed themselves, and phone numbers to call. Direct touch-dialing enabled the RE to avoid having to enter (or mis-enter) the client’s phone number. The relatively high retention rates across sites is undergoing analysis of the underlying factors. One related factor appears to be recognition by the female client of the RE who originally interviewed her and thus willingness to be re-interviewed.

### Youth RDS Survey

The Youth RDS Survey (YRDSS) was borne out of a need to measure contraceptive awareness, procurement and use among urban adolescents and youth as they enter a period of probable sexual activity. The target sample was unmarried female and male youth ages 15–24 years. Capturing this information from youth clients at health facilities, especially unmarried females, was likely to be biased due to social and familial sanctions on sexual activity and contraceptive use. In this age group, it is suspected that youth may be procuring contraceptives via other means, making their use effectively “hidden” to clinic staff and compromising the accuracy of clinic-based survey measures. Their sexual partners, relatives or other adults may be assisting with procurement. As a set of special studies, PMA Agile collaborated with youth-serving organizations in Abidjan and Nairobi, and a third has recently been launched in Lagos, to survey unmarried youth using respondent-driven sampling (RDS) methodology. This sampling method, which can be adjusted post-enumeration to weight to a known population distribution, takes advantage of youth networks for rapid recruitment and reach into diverse communities. 

The sample sizes were powered on the estimated modern contraceptive prevalence level for unmarried females 15–24 years obtained from the most recent PMA2020 survey. “Seed” respondents recruit three additional respondents, who each recruit another three until the desired sample size and gender balance, which was monitored daily, was reached. The survey was self-administered on a tablet, with an attendant RE available to guide the respondent’s use. The findings were disseminated in country once the technical report and briefs on selected topics were produced ^6^. All Agile questionnaires were translated (and back-translated) into the local languages when required. 

## Outcomes measured

PMA Agile is indicator-driven, i.e., it measures the core indicators in the service supply, quality and consumer demand environments known to influence and be of value to program officials, contraceptive and other health practices, such as commodity stock flows, client volume, client satisfaction or medication or product use adherence. Key indicators for PMA Agile are listed in Table 2, and are grouped under dimensions of supply, service quality, and demand. Additionally, it can incorporate new measures in any subsequent round of data collection desired by local stakeholders.

**Table 2.** PMA Agile data system components, indicators and health access dimension addressed.

Data unit/Indicator	Dimension
Health facility	
Provision of different FP methods	Supply
Commodity methods in/out of stock	Supply
Monthly client volume	Supply
Commodities distributed/sold in past month	Supply
Commodities received/purchased in past month	Supply
Trained providers present at time of visit	Supply
Reports data to Health Management Information System	Supply
Community outreach activities conducted	Supply
Fees charged	Supply
Provision of other Reproductive Health (RH) commodities	Supply
Provision of other Sexual and RH services (MCH, HIV)	Supply
Client	
Satisfaction with FP services/provider	Quality
Current use of contraception	
Type of method used	
Method obtained if came for FP visit	Quality
Counseled on side effects, additional methods	Quality
Provided follow-up/return information	Quality
Willingness to return/refer relative or friend	Quality
Out-of-pocket costs for FP services	Quality
Intention to use in future (for non-users)	Demand
Exposed to Behavioral Change Communications on FP	Demand
*Additional project-specific indicators are included on a site-specific basis.	

## Ethical approval

Agile data collection protocols were reviewed and approved by the Johns Hopkins Bloomberg School of Public Health Institutional Review Board and the in-country counterpart review board: Kenyatta National Hospital-University of Nairobi Ethics Research Committee (KNH-UoN ERC P470/08/2017); National Health Research Ethics Committee of Nigeria (NHREC/01/01/2007-19/09/2019); MOH-Burkina Comité d'Ethique pour la Recherche en Santé (MOH 2018-02-027); University of Kinshasa School of Public Health Institutional Review Board (ESP/CE/070/2017); Indian Institute for Health Management Research Ethical Review Board (19/12/2017-15/01-2018); MOH- Niger Comité National d'Ethique pour la Recherche en Santé (027/2020/CNERS). All participants provided consent in accordance with country specific approved consent procedures. 

## Training and data collection

### Recruitment of resident enumerators

Desired attributes of resident enumerators include: completion of secondary school, English or French literacy, local language fluency, residence in the selected city, a minimum of 21 years of age, not a paid health worker, not a health activist, no physical restrictions in conducting fieldwork, familiarity/experience with cell/smart phones, and personal awareness and support of family planning as a health intervention. In addition, preferred personal traits include maturity, self-confidence, dependability, trustworthiness, ability to protect confidentiality and respondent privacy, and social interaction skills. Recruited REs receive one week of hands-on intensive training, a smartphone and airtime.

### RE/field supervisor teams

Agile field teams were composed of six to eight interviewers and one or two field supervisors. Each city had one field team. The field supervisor assisted in the baseline selection and recruitment of SDPs to participate in the surveys. S/he also supported and oversaw the systematic sampling of clients at SDPs and their follow-up phone interviews. Each interviewer was assigned approximately 25–35 SDPs to interview each quarter depending on the geography and conducted 250–350 CEIs and another 150–200 phone interviews every six months. Field staff were salaried and retained for the entire Agile data collection period.

### Mobile phone data collection and transmittal

The collection of SDP and client data was completed with a smart mobile phone. All countries except Nigeria used JHU collect forked from the ODK collect community version 1.4.8 for the first two quarters of data collection. For all subsequent quarters of data collection, countries downloaded the latest version of the community ODK collect application as available on the Android PlayStore, prior to data collection for each quarter. Community ODK Collect versions used for data collection ranged from v1.17.1 to v1.23.3. Nigeria used the available latest versions of the application Survey CTO ranging from v2.40 to v2.60 through their 6 quarters of data collection. Nigeria also used community ODK collect v.1.17.1 and v1.25.1 to leverage its dialer app feature for the phone follow-up surveys conducted in the their 3rd and 5th quarter of data collection, respectively. Each RE was provided a basic smartphone with good functionality in Android OS (level 4.1 or higher) with adequate memory and GPS receiver having 6-meter accuracy. The smart phone had the enumeration templates to be used to record the information for each SDP. The RE uploaded each case record from the interviews to a secure cloud-based server after the interview was completed. If there was no network reception at the end of the interview, the RE stored the interview on the phone until she reached network availability and then transmitted the record to the server. 

Data are initially stored on a Google Cloud Server with access retained only by designated members of the data management and PI team. Data are downloaded off the cloud server daily to a secure server maintained either by the partner institution or the Agile project at Johns Hopkins University (JHU). Once data collection within a round was finished, all data were deleted from the cloud server and maintained only within the in-country partner’s and JHU’s systems.

**Figure 1.**
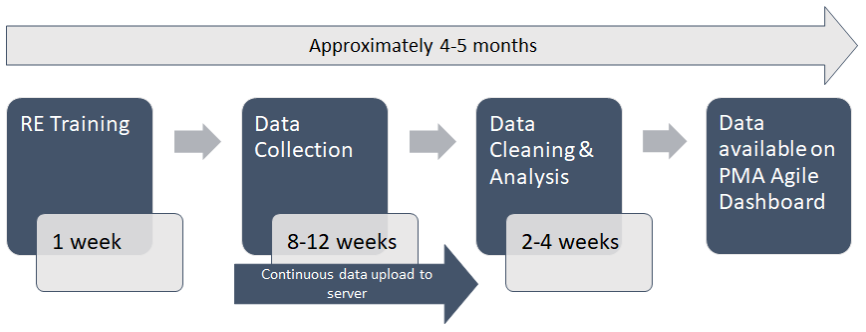
Schematic of PMA agile data collection, transmission, archiving flows.

Figure 1 illustrates the data collection and transmission cycle. A quarterly cycle can take between 11 to 17 weeks.

### Implementation schedule

**Figure 2.**
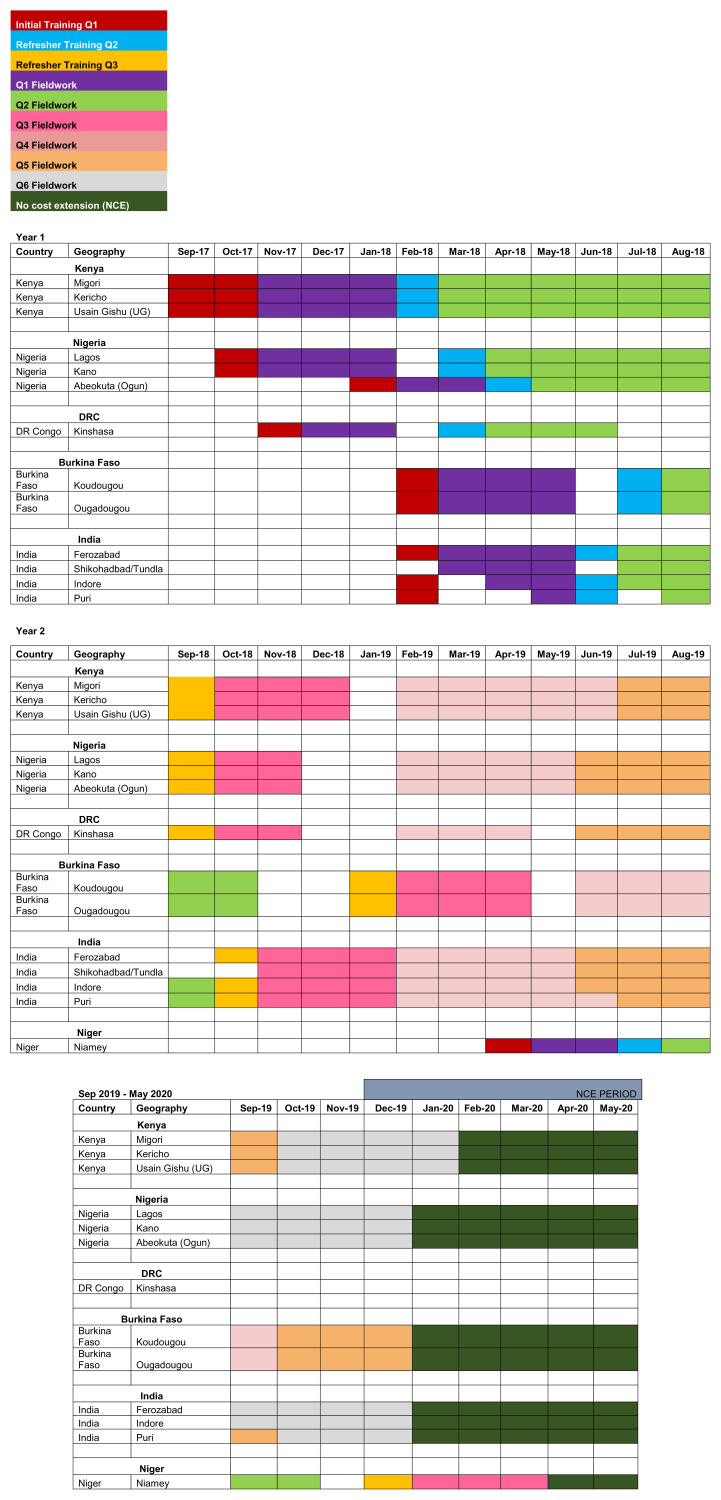
PMA Agile Data Collection Schedule.

Figure 2 provides an overview of the surveys implemented in each Agile site by year, and then in Table 3 by round and coverage of SDPs, CEIs and CEI follow-ups. The estimated population of each Agile city is also provided for context.

**Table 3.** Coverage characteristics of PMA Agile data system.

Country/Site	Population estimate LYA (000s) *	Quarter	Dates	# SDPs	# CEIs	# Female CEIs	% female	# female follow- up CEIs	% follow-up
Burkina Faso									
Ouagadougou	2,531 (2018)	1	March 2018-May 2018	212					
		2	August 2018-October 2018	205	1774	1063	59.9		
		3	February 2019-April 2019	212				876	82.4
		4	June 2019-September 2019	172	1576	886	56.2		
		5	October 2019-November 2019	191				660	74.5
Koudougou	92 (2012)	1	March 2018-May 2018	57					
		2	August 2018-October 2018	57	525	449	85.5		
		3	February 2019-April 2019	57				323	71.9
		4	June 2019-September 2019	50	449	372	82.9		
		5	October 2019-November 2019	55				263	70.7
Democratic Republic of Congo									
Kinshasa	13,171 (2018)	1	December 2017-January 2018	200					
		2	March 2018-June 2018	197	1636	1058	64.7		
		3	September 2018-November 2018	189				766	72.4
		4	February 2019-April 2019	186	1857	1219	65.6		
		5	June 2019-August 2019	184				834	68.4
India									
Ferozabad, Uttar Pradesh	604 (2011)	1	February 2018-April 2018	109					
		2	July 2018-August 2018	103	1045	505	48.3		
		3	November 2017-Jan2018	99				170	33.7
		4	February 2019-May 2019	97	967	487	50.4		
		5	June 2019-August 2019	96				305	62.6
		6	September 2019-December 2019	96	1008 (583 females)	583	57.8		
Shikohadbad and Tundla		1	February 2018-April 2018	77					
		2	July 2018-August 2018	74	737	249	33.8		
		3	November 2017-Jan2018	68				93	37.3
		4	February 2019-May 2019	68	679	285	42.0		
		5	June 2019-August 2019	68				176	61.8
Indore, Madhya Pradesh	1,994 (2011)	1	April 2018-May 2018	131					
		2	July 2018-September 2018	128	1239 (535 females)	535	43.2		
		3	November 2017-Jan2018	119				240	44.9
		4	February 2019-May 2019	114	975	492	50.5		
		5	June 2019-August 2019	110				263	53.5
		6	September 2019-December 2019	108	992	506	51.0		
Puri, Orissa	201 (2011)	1	April 2018-May 2018	97				499	98.6
		2	August 2018-October 2018	89	794	307	38.7		
		3	November 2017-Jan2018	83				156	50.8
		4	February 2019-June 2019	80	699	226	32.3		
		5	June 2019-August 2019	78				129	57.1
		6	September 2019-December 2019	75	663	213	32.1		
Kenya									
Kericho	902 (2019)	1	November 2017-Jan2018	204					
		2	March 2018-August 2018	200	1973	1439	72.9		
		3	October 2018-December 2018	198				1186	82.4
		4	February 2019-June 2019	192	1926	1307	67.9		
		5	July 2019-September 2019	202				1152	88.1
		6	October 2019-January 2020	207	2070	1255	60.6		
Migori	1,116 (2019)	1	November 2017-Jan2018	205					
		2	March 2018-August 2018	203	2011	1511	75.1		
		3	October 2018-December 2018	199				1460	96.6
		4	February 2019-June 2019	203	2030	1470	72.4		
		5	July 2019-September 2019	205				1407	95.7
		6	October 2019-January 2020	204	2040	1399	68.6		
Uasin Gishu	1,163 (2019)	1	November 2017-Jan2018	209					
		2	March 2018-August 2018	191	1858	1481	79.7		
		3	October 2018-December 2018	180				1295	87.4
		4	February 2019-June 2019	178	1750	1385	79.1		
		5	July 2019-September 2019	184				1279	92.3
		6	October 2019-January 2020	182	1810	1289	71.2		
Niger									
Niamey	1,214 (2018)	1	April 2019-June 2019	180					
		2	July 2019-Octoberr 2019	178	1012	936	92.5	n/a	n/a
		3							
Nigeria									
Kano	2,828 (2006)	1	November 2017-Jan 2018	215					
		2	March 2018-August 2018	204	1715	1202	70.1		
		3	September 2018-November 2018	203				748	62.2
		4	February 2019-May 2019	201	1816	1290	71.0		
		5	June 2019-August 2019	198				1004	77.8
		6	September 2019-November 2019	197	1780	1154	64.8		
Lagos	9,112 (2006)	1	November 2017-Jan 2018	201					
		2	March 2018-August 2018	194	1606	1294	80.6		
		3	September 2018-November 2018	191				850	65.7
		4	February 2019-May 2019	185	1487	1184	79.6		
		5	June 2019-August 2019	184				912	77.0
		6	September 2019-November 2019	179	1417	1101	77.7		
Ogun	3,751 (2006)	1	Jan 2018-March 2018	217					
		2	March 2018-August 2018	211	1707	1259	73.8		
		3	September 2018-November 2018	209				728	57.8
		4	February 2019-May 2019	202	1696	1316	77.6		
		5	June 2019-August 2019	202				933	70.9
		6	September 2019-November 2019	200	1538	1200	78.0		
Total/Average				2314	48610	33907	64.1	18707	70.2

*These population estimates are obtained from official census sources (Kenya) when possible but can be dated (Nigeria).

## Data quality monitoring, cleaning, preparation for analysis

### Data cleaning and quality monitoring

Use of ODK allows constraints and limiters to be built into the questionnaire minimizing entry errors. The date and time stamps from ODK and GPS coordinates allowed determination of the locations and times of data collection. Since these were monitored on real time basis, where unusual patterns are seen, messages for correction were sent to the team and corrective actions taken as needed.

Data cleaning checks occurred throughout data collection and after completion. Analytic routines (e.g., with Stata *.do files) were prepared and executed and generated data quality indicators that were reviewed further for outlier or illogical values by both the in-country survey IPs and the PMA Agile team at JHU. Table 4 illustrates for [Agile site] one routine for weekly monitoring of completion status for three types of Agile data. Data managers at IPs tracked progress toward reaching the sample targets on a daily basis and worked with field supervisors to trouble shoot as needed.

**Table 4.** Illustrative table of weekly survey monitoring process for Kericho in Kenya.

SDP (Quarter 4)						
Report week	Sample size	Completed	Partially completed	Refused	Not at facility/ Respondent Absent	Other
20/02/2019	8	8	0	0	0	0
27/02/2019	23	15	0	0	0	0
6/3/2019	31	8	0	0	0	0
13/03/2019	41	10	0	0	0	0
20/03/2019	54	13	0	0	0	0
27/03/2019	64	10	0	0	0	0
3/4/2019	74	10	0	0	0	0
10/4/2019	86	12	0	0	0	0
17/04/2019	91	5	0	0	0	0
24/04/2019	98	6	0	0	1	0
1/5/2019	122	20	0	0	4	0
8/5/2019	161	35	0	0	1	3
15/05/2019	166	4	0	0	1	0
22/05/2019	175	5	0	1	0	3
29/05/2019	198	7	0	5	8	3
05/06/2019	213	13	0	0	0	2
12/06/2019	223	9	0	1	0	0
19/06/2019	226	2	0	0	1	0
**Total**	****	**192**	**0**	**7**	**16**	**11**
	Client (Quarter 4)					
Report week	Sample size	Completed	Ineligible	Partially Completed	Refused	Other
20/02/2019	0	0	0	0	0	0
27/02/2019	60	60	0	0	0	0
06/03/2019	185	125	0	0	0	0
13/03/2019	365	180	0	0	0	0
20/03/2019	516	151	0	0	0	0
27/03/2019	617	99	2	0	0	0
03/04/2019	740	116	2	0	5	0
10/04/2019	828	85	0	0	3	0
17/04/2019	865	36	0	0	1	0
24/04/2019	967	98	4	0	0	0
01/05/2019	1023	56	0	0	0	0
08/05/2019	1067	44	0	0	0	0
15/05/2019	1273	204	1	0	1	0
22/05/2019	1430	151	2	0	4	0
29/05/2019	1583	153	0	0	0	0
05/06/2019	1748	159	3	0	3	0
12/06/2019	1906	157	1	0	0	0
19/06/2019	1960	51	1	0	2	0
**Total**	****	**1925**	**16**	**0**	**19**	**0**
Client follow-up (Quarter 5)						
Report week	Sample size	Completed	Participant Not available	Phone Switched off/No Answer	Wrong Number	Other
17/07/2019	182	182	0	0	0	0
24/07/2019	386	204	0	0	0	0
31/07/2019	531	145	0	0	0	0
07/08/2019	591	60	0	0	0	0
14/08/2019	645	54	0	0	0	0
21/08/2019	753	108	0	0	0	0
28/08/2019	835	82	0	0	0	0
04/09/2019	935	100	0	0	0	0
11/09/2019	993	58	0	0	0	0
18/09/2019	1070	77	0	0	0	0
25/09/2019	1131	61	0	0	0	0
02/10/2019	1148	17	1	0	0	0
09/10/2019	1152	4	7	28	7	13
**Total**		**1152**	**8**	**28**	**7**	**13**

## Data analysis

### Dashboards 

Once data files were cleaned for duplicate records, outlier or illogical values, or missing records, another set of analysis files generated the pre-selected core performance indicators, such as the proportion of SDPs reporting method-specific stockouts at the time of survey. The indicator metrics were integrated into a digital database, aka “dashboard”, which displayed the quarterly indicator data and trends therein. Public users could then view the performance statistics for the SDPs and clients on separate dashboards. Special dashboards with password access were prepared for the two large projects, TCI and DKT International. The dashboard was built such that participating SDPs could also access their own data using a unique ID provided to them. This would allow them to view their indicator data over time in relation to others in the sample (all with identities masked).

**Figure 3.**
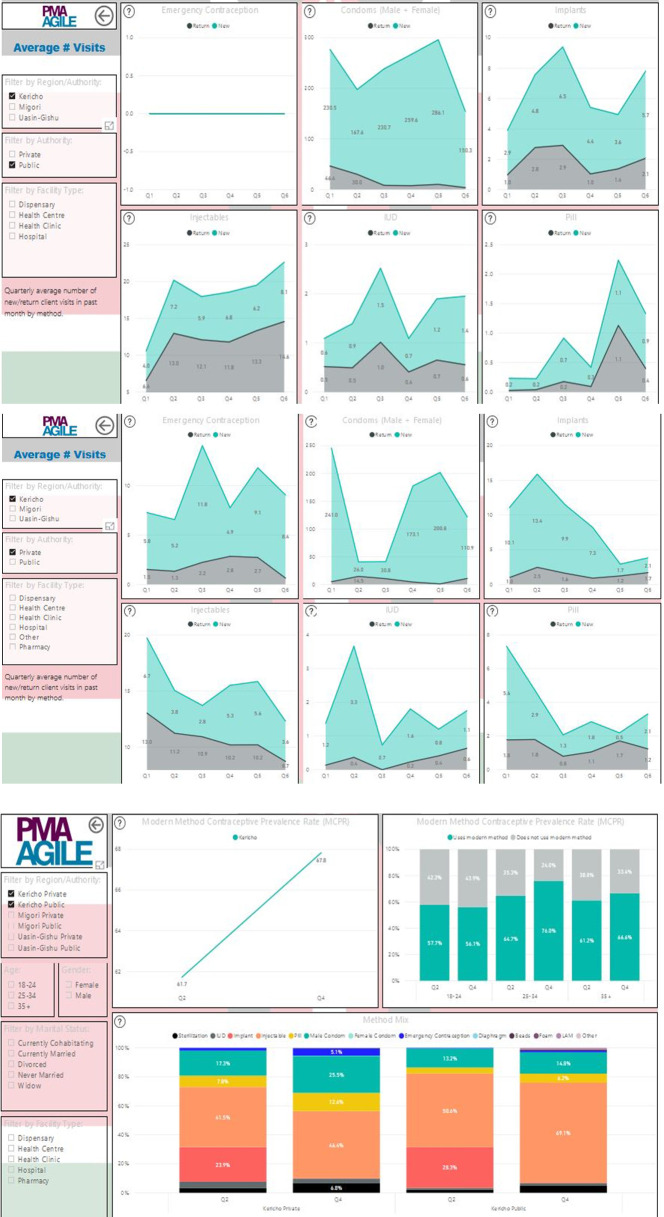
Sample screens from PMA Agile dashboard of quarterly average number of client visits by method for public and private health facilities ( **A**) and contraceptive prevalence among two rounds of client exit surveys in Kericho county, Kenya. Dashboard URL for ( **A**): https://www.pmadata.org/pma-agile-dashboard-kenya-sdp (Note that public facilities did not dispense emergency contraception at time of surveys); Dashboard URL for ( **B**): https://www.pmadata.org/pma-agile-dashboard-kenya-cei.

Figure 3A illustrates quarterly trends in one of the dashboard indicators—average number of client visits in the past month for specific contraceptive methods for urban Kericho county in Kenya among public and private SDPs (top and bottom panels respectively). Fluctuations are evident over the six quarters. Public SDPs do not dispense emergency contraception. An increasing trend in monthly client visits for injectables and higher numbers of client visits in quarters 3 and 6 for implants are visible. Tracking client visits in public facilities is indicative of demand and can be juxtaposed with stockout levels over the same quarters to assess net performance of the commodity supply chain and meeting client needs. Private consumption of contraceptives (seen in the lower panel of Figure 3A) shows more fluctuation. The average number of return client visits appears relatively stable, except for injectables, while those for new client visits is greater, especially for ECs and implants in early quarters.

Figure 3B illustrates the client indicators over two rounds of data collection in Kericho. Modern contraceptive prevalence (mCPR) is assessed among all clients as seen from the client dashboard example in Figure 3B. Although mCPR is usually measured for childbearing aged females only, the figures here are for both female and male clients, where the latter have a female partner age 15 to 49. The mCPR is 61.7% in the first round conducted in Quarter 2 and 67.8% in Quarter 4. Differences over the two quarters by client age and method mix are also shown.

The dashboards for SDPs and client indicators are publicly accessible for the 14 settings in the 6 countries at www.pmadata.org/technical-areas/pma-agile. After accessing the dashboard of interest, users can filter the indicators for each setting, public/private sector, type of facility or for clients by background characteristic (e.g., gender, age) and facility type at which the interview was conducted. 

## Data dissemination

Table 5 provides an overview of PMA Agile’s dissemination activities, which are described herein.

**Table 5.** Dissemination activities for PMA Agile.

Activity type	Objective	Frequency	URL to example	Beneficial results	Disadvantages
Stakeholder meeting	Share latest findings	Quarterly		Stakeholder reaction to findings for confirmation and actionability	Stakeholder expectation of indefinite support
PMA Agile dashboard	Provide online, real-time access to latest quarterly results	Continuous	https://www.pmadata.org/pma-agile-dashboard- kenya-sdp	Ease of referral to new stakeholders to access results; ease for updating	Access limited by weak internet connectivity in some settings
PMA Agile briefs	Share summary of indicator trends for stakeholder meetings at advanced project phases	Occasional	https://www.pmadata.org/sites/default/files/ data_product_results/PMA%20Agile-BF- Ouagadougou-Client-FR.pdf	Easy absorption of material in conventional printed format User retention of hardcopy	Labor requirements
Technical reports	Provide written summary of special data initiatives	Occasional	https://www.pmadata.org/publications/abidjan- youth-respondent-driven-sampling-survey- abidjan-yrdss-final-report	Ease of online access Focused topic	Labor requirements
Agile partner meeting	Share lessons learned across sites and updates on protocols going forward	Annual		Built network capacity and cross-site learning	
Professional conferences	Share selected findings in depth with research and practice audiences	Occasional		Opportunity to exchange insights with communities of research and practice, often visually augmented	Low priority
Journal publications	Share selected findings in depth with research and practice audiences	Occasional		Contribute to evidence base and implementation research base Publication offers permanence and archival benefits	Medium priority

### Stakeholder meetings

Agile carried out a range of dissemination activities to promote data utilization. Foremost among these were stakeholder meetings organized by the IPs usually co-sponsored with the local public health department. In addition to national and local government health officials, health staff from non-governmental and research organizations and from international projects and donor agencies were invited. An example where a close connection was forged for data utilization was the use of PMA Agile data for the family planning cost implementation plans for Kenyan counties. These stakeholder meetings also enabled local confirmation of measured and observed trends in the indicators, as was voiced in Ogun state, Nigeria, and Ouagadougou and Koudougou in Burkina Faso.

In addition to the above two main dissemination efforts, PMA Agile has produced summary briefs on SDP and client indicator trends, capturing the dashboard information, to disseminate at stakeholder meetings ^7^. These have been necessary where internet connectivity is weak and helpful in expanding knowledge about the measures and their interpretation. Technical reports are prepared for the YRDS and other special studies; and as quarterly data have accumulated, has begun a series of analyses for journal publication and conference presentations. Three annual partners meetings have been held to exchange findings, best practices and lessons learned.

## Research

PMA Agile is well-positioned to contribute to research in several areas. In particular, the project is examining topics such as: trends in stockouts among SDPs; individual and SDP factors associated with contraceptive adoption and discontinuation; the consistency of self-reported contraceptive use over time; characteristics associated with changes in contraceptive use involving traditional methods; constructing a client-based quality of care index; response patterns in CEI and CEI/Follow-up; and hidden contraceptive behaviors of youth.

## Cost

The costs of externally sponsored survey data collection efforts are often difficult to obtain and may not cover the same set of cost elements, e.g., personnel, transportation, training. Up to the time of this report, PMA Agile expended $2,736,681 on IP subawards to support personnel (central, data management, field workers), training, travel, equipment (smartphones, server), supplies, airtime, and other incidental data collection costs. Institutional indirect cost rates varied between 10 to 15%. IPs report their costs monthly enabling calculation of average costs per type of survey and over time. These are shown in Table 6. Unit costs for health facilities vary from $120 in Niger to $374 in DRC in Quarter 1 and tended to be slightly lower in Quarter 3. Client interview unit costs ranged from $9 in Niger to $40 in DRC in Quarter 2 and declined in most countries by Quarter 4. Client follow-up interviews ranged from $29 in Burkina Faso to $69 in DRC with the costs not yet known for Niger. The large sample sizes for client interviews makes them relatively cost-efficient. The total local costs for four quarters of data collection was $303,428 in Burkina Faso with two sites, $464,155 for Kinshasa, DRC, $424,550 for 3 cities in India, $1,070,938 for 3 counties in Kenya, and $1,082,773 for 3 cities in Nigeria.

**Table 6.** Estimates of PMA Agile in-country costs per data type and over four quarters.

		Unit cost ($)			
Country/Data type	# of units in baseline *	Q1 ‡	Q2	Q3	Q4
Burkina Faso					
Health Facility †	269	186	203	175	144
Client exit interview	2301		20		14
Client follow-up	1198			29	
DR Congo					
Health Facility †	200	374	371	349	365
Client exit interview	1637		40		33
Client follow-up	766			69	
India					
Health Facility †	337	274	186	170	202
Client exit interview	3077		18		18
Client follow-up	566			59	
Kenya					
Health Facility †	618	255	240	295	268
Client exit interview	5688		23		24
Client follow-up	3941			38	
Niger					
Health Facility †	180	120	90	N/A	N/A
Client exit interview	1522		9	N/A	
Client follow-up	N/A				
Nigeria					
Health Facility †	633	301	325	268	185
Client exit interview	5034		35		19
Client follow-up	2326			55	

*Baseline is defined as the first quarter each respective survey was implemented. For SDPs (Q1), for CEI baseline (Q2), for CEI follow-up (Q3). †SDP units lost to follow-up are included in the quarterly and total average costs. ‡Q1 costs include start-up/prep costs

The total PMA Agile award from the sole funding agency, The Bill & Melinda Gates Foundation, was for $4,993,285 including indirect costs of 10% for the grant period November 15, 2016 to May 31, 2020. Subawards were budgeted at $2,743,626 (63% of total direct costs). The YRDS studies ranged from $142,676 to $220,987 over the three sites and are not included in the unit cost calculations. 

## Discussion

Establishing and maintaining a well-functioning M&E system that routinely collects data on the supply chain systems and management ( Mukasa *et al*., 2017) to report on the performance of public and private family planning programs plays a critical role in addressing gaps in access to contraceptive information and services in low- and middle-income countries (LMICs). In this paper, we described the design, organization and implementation of a reliable and standardized M&E system that regularly collected, linked and aggregated data at different levels on a focused set of indicators in a cost-effective manner. Facility and client data can be linked, enabling an appreciation of the consumer environment (see Ahmed *et al.*, 2014; Larson *et al.*, 2019). The system has made a needed contribution in rapidly producing survey estimates of the indicators at a sub-national level using mobile phone technology and a dedicated small team of enumerators and supervisors.

The PMA Agile platform demonstrated it is possible to regularly collect data on over 2300 Service Delivery Points (both public and private) to track client volume and commodity sales and stock outs. It interviewed nearly 34,000 female and 16,000 male clients of childbearing age irrespective of their current contracepting status and reached over 18,700 of the female clients for follow-up phone interviews, across 14 urban settings in six countries, Burkina Faso, DRC, India, Kenya, Niger and Nigeria and within a 26-month timeframe. The platform also rapidly posted indicator data on 14 publicly accessible dashboards at the SDP and client levels for stakeholders to view and monitor program progress. To understand procurement of contraceptive supplies by unmarried young persons, three youth respondent-driven sampling surveys in three major cities were also conducted. By design, the PMA Agile system leveraged stakeholder engagement early in planning, implementing and monitoring family planning services. It provided valuable learning tools for health workers and less expensive means for program managers to obtain local and actionable information to improve city services. At stakeholder dissemination events, local public health providers and officials often confirmed the results’ profiles as aligning with their own perceptions.

Despite its innovations and strengths, PMA Agile also encountered implementation challenges. In several geographies, the local teams had to resolve issues related to incomplete master SDP lists. Facilities were found to be not operational, closed, or their addresses had changed by the time of the survey. In a few settings the distinction between a public or private SDP was blurred in practice. The systematic sampling of clients in advanced facilities often required a second enumerator, where client volume could slow completion of interviews and where otherwise casual interviewing could incur other types of information bias, selection bias and sampling error ( Eisele *et al.*, 2013). One critical perspective missing from PMA Agile’s provision of a total assessment of the health system’s performance in family planning is that of providers. Their interactions with clients, counseling skills and technical competence are important to evaluate ( Hutchinson *et al.*, 2011; Solo & Festin, 2019) and could be added to the platform on a regular basis. Resources permitting, this addition could be a useful means to assess human resource needs.

The PMA Agile platform was designed to be replicable, expandable and adaptable obtaining data to scale with the potential to be linked with population, spatial, administrative and other types of information for district-level planning. It has been implemented following standardized protocols with strict quality control across all aspects of sample selection, data collection, analysis and dissemination. Ideally external data systems should complement publicly established ones and not duplicate effort or require new resources. However, in the case of family planning, the quality of LMIC health information systems data have typically been weak and confined to government facilities. Since considerable contraceptive care is obtained from private providers, neglecting measurement of this sector’s contribution can significantly bias the understanding where access gaps exist. The PMA Agile platform can also support implementation research and as such, its potential will hopefully be exploited in the coming years.

## Data availability

De-identified data from PMA Agile are publicly available from each individual country. To request PMA Agile data, please email the relevant country-specific address: Burkina Faso Agile Data Request burkinafaso.agile.data@pma2020.org; DRC Agile Data Request drc.agile.data@pma2020.org; India Agile Data Request india.agile.data@pma2020.org; Kenya Agile Data Request kenya.agile.data@pma2020.org; Niger Agile Data Request niger.agile.data@pma2020.org; Nigeria Agile Data Request nigeria.agile.data@pma2020.org.

There are no restrictions on who can apply to access the data. Those interested in using the data will be asked to complete a form that includes the purpose of the analysis, and confirmation of various data use considerations. 

## Notes

^1^ Agile retains the core innovation of PMA (formerly PMA2020), where women are recruited from or near the selected enumeration area and trained to collect data using smartphones on a repeated and quick-turnaround basis ( Zimmerman *et al.*, 2017). 

^2^ At the time, the first of the two projects was these were The Challenge Initiative, and urban-focused family planning initiative, located in the Bill & Melinda Gates Institute for Population and Reproductive Health at Johns Hopkins Bloomberg School of Public Health (tciurbanhealth.org), the second and was an expansion social marketing project implemented by DKT International (dktinternational.org).. Both foundation investments were initiated in 2016.

^3^ Questionnaires for each can be accessed at https://pmadata.org/technical-areas/pma-agile. 

^4^ We use health facility and SDP interchangeably.

^5^ In India the number of urban primary health centers was very small in each site. The private sector sample size was accordingly increased.

^6^ These can be accessed on the PMA Agile webpage, for example https://www.pmadata.org/sites/default/files/2019-07/English_CI-YRDSS_Report_FINAL.pdf

^7^ An example of the Burkina Faso SDP brief can be accessed at https://www.pmadata.org/sites/default/files/data_product_results/PMA%20Agile-BF-Ouagadougou-SDP-French2.pdf. A client indicator brief is also available in English and French as well.

## References

[ref-1] Ahmed F Burt J Roland M: Measuring patient experience: concepts and methods. *Patient.* 2014;7(3):235–41. 10.1007/s40271-014-0060-5 24831941

[ref-2] Boerma JT Sommerfelt AE: Demographic and health surveys (DHS): contributions and limitations. *World Health Stat Q.* 1993;46(4):222–226. 8017081

[ref-3] Braa J Hanseth O Heywood A: Developing health information systems in developing countries: The flexible standards strategy. *MIS Q.* 2007;31(2):381–402. 10.2307/25148796

[ref-4] Eisele TP Rhoda DA Cutts FT: Measuring coverage in MNCH: total survey error and the interpretation of intervention coverage estimates from household surveys. *PLoS Med.* 2013;10(5):e1001386. 10.1371/journal.pmed.1001386 23667331 PMC3646211

[ref-5] Guenther T Lainez YB Oliphant NP: Routine monitoring systems for integrated community case management programs: Lessons from 18 countries in sub-Saharan Africa. *J Glob Health.* 2014;4(2):020301. 25520787 10.7189/jogh-04-020301 PMC4267095

[ref-6] Hutchinson PL Do M Agha S: Measuring client satisfaction and the quality of family planning services: a comparative analysis of public and private health facilities in Tanzania, Kenya and Ghana. *BMC Health Serv Res.* 2011;11:203. 10.1186/1472-6963-11-203 21864335 PMC3224259

[ref-7] Larson E Sharma J Bohren MA: When the patient is the expert: measuring patient experience and satisfaction with care. *Bull World Health Organ.* 2019;97(8):563–569. 10.2471/BLT.18.225201 31384074 PMC6653815

[ref-8] Maina I Wanjala P Soti D: Using health-facility data to assess subnational coverage of maternal and child health indicators, Kenya. *Bull World Health Organ.* 2017;95(10):683–694. 10.2471/BLT.17.194399 29147041 PMC5689197

[ref-9] Mukasa B Ali M Farron M: Contraception supply chain challenges: a review of evidence from low- and middle-income countries. *Eur J Contracept Reprod Health Care.* 2017;22(5):384–390. 10.1080/13625187.2017.1394453 29087737

[ref-10] Nordberg E: Household health surveys in developing countries: Could more use be made of them in planning? *Health Policy Plan.* 1988;3(1):32–39. 10.1093/heapol/3.1.32

[ref-11] Rowe AK: Potential of integrated continuous surveys and quality management to support monitoring, evaluation, and the scale-up of health interventions in developing countries. *Am J Trop Med Hyg.* 2009;80(6):971–979. 10.4269/ajtmh.2009.80.971 19478260

[ref-12] Solo J Festin M: Provider Bias in Family Planning Services: A Review of Its Meaning and Manifestations. *Glob Health Sci Pract.* 2019;7(3):371–385. 10.9745/GHSP-D-19-00130 31515240 PMC6816811

[ref-15] United Nations, Department of Economic and Social Affairs, Population Division: World Urbanization Prospects: The 2018 Revision (ST/ESA/SER.A/420). New York: United Nations,2019 Reference Source

[ref-13] Walsham G Sahay S: Research on information systems in developing countries: Current landscape and future prospects. *Inform Technol Dev.* 2006;12(1):7–24. 10.1002/itdj.20020

[ref-14] Zimmerman L Olson H, PMA2020 Principal Investigators Group: PMA2020: Rapid Turn-Around Survey Data to Monitor Family Planning Service and Practice in Ten Countries. *Stud Fam Plann.* 2017;48(3):293–303. 10.1111/sifp.12031 28885679 PMC6084342

