# Supplementary material for: Performance monitoring and accountability: The Agile Project’s protocol, record and experience
Source: Gates Open Res. 2020 Aug 20;4:30. Originally published 2020 Mar 25. [Version 2] doi: 10.12688/gatesopenres.13119.2 (PMC7463111; doi:10.12688/gatesopenres.13119.2)
Supplement: Peer review history

# Peer review: Reviewer response for version 2

**DOI:** 10.21956/gatesopenres.14359.r29415

**Published:** 2020-09-01

**Authors:** Kristin Bietsch, Emily Sonneveldt

**Affiliations:** 1 Track20 Project, Avenir Health, Glastonbury, CT, USA; 2 Avenir Health, Glastonbury, CT, USA

We appreciate the authors’ detailed responses and approve this article for publication.

Is the study design appropriate for the research question?

Yes

Is the rationale for, and objectives of, the study clearly described?

Yes

Are sufficient details of the methods provided to allow replication by others?

Partly

Are the datasets clearly presented in a useable and accessible format?

Not applicable

Reviewer Expertise:

Family Planning, Monitoring and Evaluation

We confirm that we have read this submission and believe that we have an appropriate level of expertise to confirm that it is of an acceptable scientific standard.

# Peer review: Reviewer response for version 2

**DOI:** 10.21956/gatesopenres.14359.r29416

**Published:** 2020-08-21

**Authors:** Wanapa Naravage

**Affiliations:** 1 Faculty of Public Health, Thammasat University, Khlong Luang, Pathumthani, Thailand

I would like to confirm that I read the revised version and approved it.

Is the study design appropriate for the research question?

Yes

Is the rationale for, and objectives of, the study clearly described?

Yes

Are sufficient details of the methods provided to allow replication by others?

Yes

Are the datasets clearly presented in a useable and accessible format?

Yes

Reviewer Expertise:

NA

I confirm that I have read this submission and believe that I have an appropriate level of expertise to confirm that it is of an acceptable scientific standard.

# Peer review: Reviewer response for version 1

**DOI:** 10.21956/gatesopenres.14298.r28828

**Published:** 2020-06-03

**Authors:** Wanapa Naravage

**Affiliations:** 1 Faculty of Public Health, Thammasat University, Khlong Luang, Pathumthani, Thailand

I am pleased to provide my comments for the study entitled “Performance monitoring and accountability: The Agile Project’s protocol, record and experience" as follows. In general, the study by Angelwicz *et al.* give an interesting multi-site study on the performance monitoring and accountability (PMA). The main contribution of the paper offers an excellent opportunity for stakeholders for tracking and acting on performance results on an ongoing of the two large projects. Also, the protocol provides enough details for reader to repeat the PMA. However, I have some additional comments about the paper listed below:

1. Introduction: On page 3 of 19, the last paragraph on the left, it is stated that “...innovative data system that could track performance of two large projects…” I would recommend that the paper should provide more information on the key inputs of family planning innovations as it will allow replication by others if they have the similar interventions. In addition, it would be helpful if the paper provides information on when the two large projects start.
2. Protocol: On page 3 of 19, the first paragraph “…to 70 percent by 2050.” Please provide the reference.
3. CEI Follow-up: On page 5 of 19, the first paragraph, “…, only female clients were recruit.” In the client exit interview both women and men were recruited, but at the CEI follow up, only women were recruited. Please provide rationale why men were not recruited.
  In addition, please provide additional information of the gender of interviewers who do the client exit interview and follow-up telephone interview. As contraceptive methods are a bit sensitive when talking unlike other products, women may feel uncomfortable to disclose their practice about contraceptive practice with a male interviewer.
4. Ethical approval: On page 5 of 19, please provide the ethical approval number of each study site.

Thank you for your kind consideration.

Is the study design appropriate for the research question?

Yes

Is the rationale for, and objectives of, the study clearly described?

Yes

Are sufficient details of the methods provided to allow replication by others?

Yes

Are the datasets clearly presented in a useable and accessible format?

Yes

Reviewer Expertise:

Sexual and reproductive health, family health, contraception and health systems.

I confirm that I have read this submission and believe that I have an appropriate level of expertise to confirm that it is of an acceptable scientific standard.

# Response

**Published:** 2020-07-16

**Authors:** Philip Anglewicz

**Affiliations:** Johns Hopkins Bloomberg School of Public Health, USA

We thank this reviewer for the useful comments. Below we list each comment, followed by our response:

Response to GOR Review #2 on Agile protocol:

I am pleased to provide my comments for the study entitled “Performance monitoring and accountability: The Agile Project’s protocol, record and experience" as follows. In general, the study by Tsui et al. give an interesting multi-site study on the performance monitoring and accountability (PMA). The main contribution of the paper offers an excellent opportunity for stakeholders for tracking and acting on performance results on an ongoing of the two large projects. Also, the protocol provides enough details for reader to repeat the PMA. However, I have some additional comments about the paper listed below:

*We appreciate the positive comments about this project.*

Introduction: On page 3 of 19, the last paragraph on the left, it is stated that “...innovative data system that could track performance of two large projects…” I would recommend that the paper should provide more information on the key inputs of family planning innovations as it will allow replication by others if they have the similar interventions. In addition, it would be helpful if the paper provides information on when the two large projects start.

*Just to confirm that our characterization of the Agile data system as being “innovative” pertains to data collection as opposed to “family planning innovations”, such as delivery interventions or contraceptive development advances.  The two large projects were themselves innovative and we have included links to their websites for interested readers to explore, along with the start year (2016).*

Protocol: On page 3 of 19, the first paragraph “…to 70 percent by 2050.” Please provide the reference.

*We have added a reference for this statement.*

CEI Follow-up: On page 5 of 19, the first paragraph, “…, only female clients were recruit.” In the client exit interview both women and men were recruited, but at the CEI follow up, only women were recruited. Please provide rationale why men were not recruited.

*Male clients could have been re-interviewed but we did not pursue this for the following reasons:*

*1)    Male clients accounted for about 1 in 3 clients interviewed. The project’s resources were constrained and we were not sure how much effort would be required to successfully reach male clients.  In total we interviewed nearly 33.9 thousand female clients and of them about 18.7 thousand were successfully interviewed for the follow-up (70%).  If we had the resources, we would have endeavored to recontact the male clients.*

*2)    The focus of the follow-up was to measure contraceptive continuation, switching or adoption.  Generally speaking, this information is most accurately obtained from the female partner.*

In addition, please provide additional information of the gender of interviewers who do the client exit interview and follow-up telephone interview. As contraceptive methods are a bit sensitive when talking unlike other products, women may feel uncomfortable to disclose their practice about contraceptive practice with a male interviewer.

*PMA Agile only used female interviewers for the SDP, CEI, and CEI follow up interviews.*

Ethical approval: On page 5 of 19, please provide the ethical approval number of each study site.

*The ethical approval numbers are as follows, we have added this to the “ethical approval” section of the paper on pg 10:*

*Burkina Faso:  Ministry of Health No. 2018-02-027*

*Nigeria:  NHREC/01/01/2007-19/09/2019*

*India:  IIHMR 19/12/2017-15/01-2018*

*Niger:  Ministry of Health No. 027/2020/CNERS*

*Kenya:  KNH-UoN ERC P470/08/2017*

*DRC:  University of Kinshasa No. ESP/CE/070/2017*

# Peer review: Reviewer response for version 1

**DOI:** 10.21956/gatesopenres.14298.r28719

**Published:** 2020-05-19

**Authors:** Kristin Bietsch, Emily Sonneveldt

**Affiliations:** 1 Avenir Health, Glastonbury, CT, USA; 2 Track20 Project, Avenir Health, Glastonbury, CT, USA

This project offers a 4-prong approach to studying health facilities in urban areas in developing countries. It uses electronic equipment including smart phones and tablets to aid in follow up and discretion. We enjoyed reading this paper and look forward to the research produced by this data collection. Answers to the questions below would give readers a greater understanding of the data collection and implications of this project: 

**General Questions and Comments: **

- Overall, the paper does an excellent job of describing the overall process used to collect the various types of data. However, it would be helpful to add more detail about the calculations used to produce indicators. Many of the questions in the next section are aimed at identifying specific additions to the methodology section.
- This project focuses on urban areas - what would stay the same and would change for this methodology to work in rural areas or nationally?
- It is assumed that there is already a lot of research using this data that is already underway, but can you add a section discussing what type of research this data lends itself to and how it has been used to monitor the two projects discussed in footnote 2?
- For the cost information, it would be helpful to know the relationship to overall costs to the sub-contracted costs that are used to determine the costs of specific data collection methods. Without this information it is not possible to determine if the approach is cost effective or not.
- Looking at Table 2, it would be nice, since this is a survey methodology paper, to have a discussion of the other methods of data collection (HMIS, LMIS, SPA, UNFPA facility surveys, etc.) and the added value of PMA Agile.  A cost comparison, though it may be difficult to collect cost information from others, would be very interesting. Additionally, you can add lessons learned from PMA Agile and what you would suggest governments add to their routine data collection.
  - Are there sustainability plans in place with countries to transfer some of this data collection to routine monitoring already done at the facility level by countries?
- The relatively high retention rate for CEI follow ups is an interesting finding - hopefully this can be used as motivation of other surveys to use cell phones for follow up and will allow for more interesting and affordable panel data!

**Questions about Sampling and Methodology:**

 Overall, it would be helpful to have more detail about your sampling methodology, including weighting. Specifically:

- Your CEI calculations are based off an assumption of 50% mCPR - is this for married or all women? Can you give the range of mCPR in your cities?
- In your CEI, you interview both males and females - are they given the same questionnaire? Are they from the same sample (interviewing 1 out of x number of people who leave the facility)? Can you talk more about the motivation to include men in your sample and what you hope to learn from these interviews? What were the sample sizes of men in your cities?
- Do you have weights in place given that you have the same number of public and private facilities but there are probably not an even number of private and public facilities in the city?
- On page 5, can you explain the difference between participation rates and non-consent?
- What was the thinking behind the even split among public and private facilities?
  - Across your sites, did you have information on the share of contraceptive users that receive methods from public versus private when creating your sampling frame?
  - What are the margins of error when looking at only results for public or private facilities?
- On page 4, how do the other countries compare to the 77% of SDPs in Kenya that provide 3 or more methods?
- Table 1: Do you have estimates on the scale of non-registered facilities and what proportion of contraceptive users receive their methods from them? Also, what is the turnover of registered health facilities - how often would a longer-term project need to refresh the sampling frame?
- In general, can you elaborate on weights? Are large clinics and small clinics weighted the same? Or are there weighting options based off the size of the population they serve? Having these weights would allow you to look at both the facility and women as the unit of interest. For example, you could then report both the percentage of facilities with stockouts and the percentage of women who go to a facility with a stockout.
- Do you apply weights to the CEI interviews? If so, what information is used to create the weights?
- Since the geographical area covered by data collection does not appear to necessarily be a standard administrative delineation, it would be helpful to have additional information on how denominators are constructed. Are they combined administrative units? Are they based on facility catchment areas?

**Questions Related to mCPR Calculations:** **Questions Related to Specific Figures:**

- Figure 3A: outlining the two panels in black boxes would help distinguish them as two different graphics.
- Figure 3B: having the top left panel axis not extend to 0 is misleading.

For the dashboards, it may be helpful to uses dates instead of Q’s, or at least include years.

Is the study design appropriate for the research question?

Yes

Is the rationale for, and objectives of, the study clearly described?

Yes

Are sufficient details of the methods provided to allow replication by others?

Partly

Are the datasets clearly presented in a useable and accessible format?

Not applicable

Reviewer Expertise:

Family Planning, Monitoring and Evaluation

We confirm that we have read this submission and believe that we have an appropriate level of expertise to confirm that it is of an acceptable scientific standard, however we have significant reservations, as outlined above.

# Response

**Published:** 2020-07-16

**Authors:** Philip Anglewicz

**Affiliations:** Johns Hopkins Bloomberg School of Public Health, USA

We appreciate the comments from these reviewers, as they have helped to improve the manuscript.  We list our responses below each comment:

Response to GOR Review #1 on Agile protocol:

This project offers a 4-prong approach to studying health facilities in urban areas in developing countries. It uses electronic equipment including smart phones and tablets to aid in follow up and discretion. We enjoyed reading this paper and look forward to the research produced by this data collection. Answers to the questions below would give readers a greater understanding of the data collection and implications of this project: 

General Questions and Comments: 

•    Overall, the paper does an excellent job of describing the overall process used to collect the various types of data. However, it would be helpful to add more detail about the calculations used to produce indicators. Many of the questions in the next section are aimed at identifying specific additions to the methodology section.

*The exact calculation for each indicator is available on the project dashboard site (https://www.pmadata.org/pma-agile-dashboard-kenya-cei).  On each page of the dashboard there is a circled question mark; when the cursor is moved to this question mark, the calculation of the indicator appears.*

•    This project focuses on urban areas - what would stay the same and would change for this methodology to work in rural areas or nationally?

*A rural application of the same sampling methodology would only require ensuring access to a complete listing of public and private health facilities, stratifying by the same broad facility types, and selection a number that is adequately powered and proportional to the rural: urban distribution and within facility strata.  Appropriate weighting would be needed but together with the urban data would generate national estimates.*

*Ideally the client samples will also have weights to generate representative estimates. Telephone follow-up would depend on phone access or other means of timely re-interview.*

•    It is assumed that there is already a lot of research using this data that is already underway, but can you add a section discussing what type of research this data lends itself to and how it has been used to monitor the two projects discussed in footnote 2?

*We have added a paragraph on research to the “Data dissemination” section of the paper. The project is indeed working on several research papers at present, including the following topics:*

*-    Trends in stockouts among Agile SDPs.*

*-    Individual and SDP factors associated with contraceptive adoption and discontinuation.*

*-    The consistency of self-reported contraceptive use over time.*

*-    Characteristics associated with changes in contraceptive use involving traditional methods in DRC.*

*-    Constructing a client-based quality of care index.*

*-    Response patterns in CEI and CEI/Follow-up.*

*-    Hidden contraceptive behaviors of youth.*

*Insofar as use of the data for the two projects, we established separate dashboards for the project staff at TCI and DKT International to self-monitor.*

•    For the cost information, it would be helpful to know the relationship to overall costs to the sub-contracted costs that are used to determine the costs of specific data collection methods. Without this information it is not possible to determine if the approach is cost effective or not.

*We provided the total amount of the project grant ($4,993,285) which is still ongoing.  Less 10% indirect costs, the direct cost amount is $4,539,350.  Less the $2,743,626 budgeted to partner subawards, the direct costs remaining for Baltimore technical support and administration is $1,795,724 (40%).*

*We did not distribute the central costs by country.  Central technical support costs included data collection design and protocols, data processing and management, dashboard implementation and data analysis. As quarterly data collection was repeated, technical support costs per cycle have reduced. Research costs to publish were only supported in recent months under the no-cost extension.*

•    Looking at Table 2, it would be nice, since this is a survey methodology paper, to have a discussion of the other methods of data collection (HMIS, LMIS, SPA, UNFPA facility surveys, etc.) and the added value of PMA Agile.  A cost comparison, though it may be difficult to collect cost information from others, would be very interesting. Additionally, you can add lessons learned from PMA Agile and what you would suggest governments add to their routine data collection. 

*Because this is a protocol paper, the type of information gathering and review suggested – which we agree would indeed be very valuable and a major undertaking – is beyond the scope of the present effort.*

o    Are there sustainability plans in place with countries to transfer some of this data collection to routine monitoring already done at the facility level by countries?

*There was a dedicated effort to integrate the Agile Nigeria data collection into the Federal Ministry of Health’s family planning dashboard at the facility level.  This was precluded by an IRB condition not to disclose facility identities.  Other challenges would have been alignment on type and measurement of indicators and dates of data collection.  In every location, however, Agile results have been disseminated to the relevant local government and non-government stakeholders, usually through in-person meetings.*

•    The relatively high retention rate for CEI follow ups is an interesting finding - hopefully this can be used as motivation of other surveys to use cell phones for follow up and will allow for more interesting and affordable panel data!

*Agree!*

Questions about Sampling and Methodology:

Overall, it would be helpful to have more detail about your sampling methodology, including weighting. Specifically: 

•    Your CEI calculations are based off an assumption of 50% mCPR - is this for married or all women? Can you give the range of mCPR in your cities?

*The CEI client sample was powered with consideration to the male/female composition and overall daily client volume (wherein we consulted the PMA2020 SDP data), rather than the mCPR. No constraint on marital status was used.  We do not have a range of mCPRs for the sites but we publish on the dashboard the % of clients who reporting currently using modern contraception.*

•    In your CEI, you interview both males and females - are they given the same questionnaire? Are they from the same sample (interviewing 1 out of x number of people who leave the facility)? Can you talk more about the motivation to include men in your sample and what you hope to learn from these interviews? What were the sample sizes of men in your cities?

*Yes, the same questionnaire is used for male and female clients.  Yes, clients are selected without regard to gender.  The client samples are usually 10 times the number of facilities.  Men comprise approximately 30% of these across all sites (see Table 2 bottom).  Because males often purchase condoms for use with female partners, we wanted to capture their consumption and use behaviors.*

•    Do you have weights in place given that you have the same number of public and private facilities but there are probably not an even number of private and public facilities in the city?

*Yes the public access data files have SDP (facility) weights.  The sampling protocol and weight construction can be obtained at the following site: https://www.pmadata.org/sites/default/files/2020-06/PMA%20Agile%20Sampling%20and%20Weighting%20Protocol.pdf*

•    On page 5, can you explain the difference between participation rates and non-consent?

*Participation rates and non-consent mean the same thing: the percentage of clients that consented to be interviewed.  We have clarified this point in the text.*

•    What was the thinking behind the even split among public and private facilities? 

*It was to ensure an adequate number of each type.  It was not always 100+100 because a number of Agile sites were small and had less than the target number.  If less than 100, the full census of available facilities was conducted.  We also oversampled the SDP sample by 10%, so 220 facilities, to allow for attrition over time. The facility weight takes into account the public-private distribution and the oversampling.*

•    Across your sites, did you have information on the share of contraceptive users that receive methods from public versus private when creating your sampling frame?  

*No.  This was also not available in the larger national surveys which did not cover Agile’s urban sites.*

•    What are the margins of error when looking at only results for public or private facilities?  

*This would depend on the indicator of interest.  It can be generated using most statistical software.  For example, the 95%CI around the mean proportion offering family planning by 312 public SDPs in Kenya quarter 1 of 0.984 is 0.970-0.998.*

•    On page 4, how do the other countries compare to the 77% of SDPs in Kenya that provide 3 or more methods?

*We recommend examining the dashboards or obtaining the public access data files to answer questions regarding values for the data collected.*

•    Table 1: Do you have estimates on the scale of non-registered facilities and what proportion of contraceptive users receive their methods from them? Also, what is the turnover of registered health facilities - how often would a longer-term project need to refresh the sampling frame?

*Because non-registered facilities are not in a registry, they could not be included as a sampling frame for this project.  This is a selection issue primarily for Nigeria sites where patent medical vendors (PMVs) are plentiful and often the source of short-term contraceptives.  Kiosks in India are also excluded.  Conducting a client exit interview at these outlets would have been a significant challenge.*

*We do not know how much turnover happens over multiple years.  Over the six quarters of data collection in the longest-duration sites, you can surmise turnover from Table 2, looking at the change between Q1 and Q6.  A longer-term project should probably repeat the mapping at least every second year to assess change in SDP availability and retention of sample power.*

•    In general, can you elaborate on weights? Are large clinics and small clinics weighted the same? Or are there weighting options based off the size of the population they serve? Having these weights would allow you to look at both the facility and women as the unit of interest. For example, you could then report both the percentage of facilities with stockouts and the percentage of women who go to a facility with a stockout.

*See the above bullet response regarding obtaining more information on weight construction.  Clinics are weighted by facility group type which generally correspond to size (from hospitals down to urban health posts).  Yes, you can link the client data to the facility where s/he was interviewed but for now, we cannot weight the Agile client data (see next bullet).*

Do you apply weights to the CEI interviews? If so, what information is used to create the weights?

*No.  Because the REs did not uniformly record the total number of clients approached, we do not have a daily selection probability which would be needed to weight the client data.  A forthcoming analysis describes response rates for the client data.*

•    Since the geographical area covered by data collection does not appear to necessarily be a standard administrative delineation, it would be helpful to have additional information on how denominators are constructed. Are they combined administrative units? Are they based on facility catchment areas?

*The geographic coverage is the administrative boundaries for the urban cities in Burkina Faso, Niger, India, and DR Congo.  For Kenya, it is the urban area of the 3 counties.  For Nigeria it is the boundaries of Lagos and Kano city states and the urban are of Ogun State.  For health facilities, the denominators are those within the administrative boundaries.  No catchment areas were used for sampling.*

Questions Related to mCPR Calculations: Questions Related to Specific Figures: 

•    Figure 3A: outlining the two panels in black boxes would help distinguish them as two different graphics.

*We agree and have added black outlines to each panel.*

•    Figure 3B: having the top left panel axis not extend to 0 is misleading.

*For all of the graphs on the PMA Agile dashboard, we scale the axis to fit the range of numbers included, and generally don’t extend the axis to numbers out of range.  Although this may appear to exaggerate changes, as described in this comment, it also makes the results easier to read.  To reduce misinterpretation and guide the viewer on the extent of change over time, all values appear on the graphs.*

  

•    For the dashboards, it may be helpful to uses dates instead of Q’s, or at least include years.

*We appreciate this point. After testing the dashboard with dates, we found that, in nearly all cases, the graph labels are not readable if dates (even just years) are added. This is partly because the Q’s sometimes straddled years (between 2018 and 2019). However, we note that the timing and duration of each Q is presented in slide 2 in all dashboards, as well as Table 3 and Figure 2 in this paper.*
